# Artificial Intelligence and Machine Learning Applications in Sudden Cardiac Arrest Prediction and Management: A Comprehensive Review

**DOI:** 10.1007/s11886-023-01964-w

**Published:** 2023-10-04

**Authors:** Sarah Aqel, Sebawe Syaj, Ayah Al-Bzour, Faris Abuzanouneh, Noor Al-Bzour, Jamil Ahmad

**Affiliations:** 1 Medical Research Center, Hamad Medical Corporation, Doha, Qatar; 2 Faculty of Medicine, Jordan University of Science and Technology, Irbid, Jordan; 3 Department of Urology, Hamad Medical Corporation, Doha, Qatar

**Keywords:** Sudden cardiac arrest, Artificial intelligence, Machine learning, Prediction models, Cardiopulmonary resuscitation

## Abstract

**Purpose of Review:**

This literature review aims to provide a comprehensive overview of the recent advances in prediction models and the deployment of AI and ML in the prediction of cardiopulmonary resuscitation (CPR) success. The objectives are to understand the role of AI and ML in healthcare, specifically in medical diagnosis, statistics, and precision medicine, and to explore their applications in predicting and managing sudden cardiac arrest outcomes, especially in the context of prehospital emergency care.

**Recent Findings:**

The role of AI and ML in healthcare is expanding, with applications evident in medical diagnosis, statistics, and precision medicine. Deep learning is gaining prominence in radiomics and population health for disease risk prediction. There’s a significant focus on the integration of AI and ML in prehospital emergency care, particularly in using ML algorithms for predicting outcomes in COVID-19 patients and enhancing the recognition of out-of-hospital cardiac arrest (OHCA). Furthermore, the combination of AI with automated external defibrillators (AEDs) shows potential in better detecting shockable rhythms during cardiac arrest incidents.

**Summary:**

AI and ML hold immense promise in revolutionizing the prediction and management of sudden cardiac arrest, hinting at improved survival rates and more efficient healthcare interventions in the future. Sudden cardiac arrest (SCA) continues to be a major global cause of death, with survival rates remaining low despite advanced first responder systems. The ongoing challenge is the prediction and prevention of SCA. However, with the rise in the adoption of AI and ML tools in clinical electrophysiology in recent times, there is optimism about addressing these challenges more effectively.

## Introduction

Sudden cardiac arrest (SCA) remains a prominent global cause of mortality comprising 15–20% of all deaths worldwide, and accounting for 50–100 cases per 100,000 individuals [1••, 2]. Cardiopulmonary resuscitation (CPR) administration and the use of electrical defibrillation are crucial interventions to achieve effective resuscitation in patients experiencing ventricular fibrillation (VF) rhythms and cardiac arrest [3]. Despite the existence of advanced first responder systems for cardiac arrest resuscitation, a recent analysis in North America revealed a meager overall survival rate of 4.6%. Unfortunately, a significant portion of administered shocks remain unsuccessful in achieving the return of spontaneous circulation [4, 5].

Several demographic, clinical, environmental, and genetic factors affect the incidence and survival rates of SCA victims. As for adults, SCA incidence rates are linked with increased age, while survival rates tend to be worse and higher death rates are seen among younger age groups [6, 7]. The prediction and prevention of SCA constitute significant challenges that hinder the effectiveness and cost–benefit of existing methodologies [8]. The utilization of artificial intelligence (AI) tools is on the rise for tackling intricate issues, and they are well-positioned to address the significant unmet requirement in the field of clinical electrophysiology. Consequently, AI tools have become crucial to differentiate the shockable subgroup of sudden cardiac arrest (SCA). There is an urgent demand to discover novel predictors of SCA in individuals [9]. Thus, here we aimed to review the recent advances in prediction models and the deployment of AI and machine learning (ML) in the prediction of CPR success.

### Role of AI and ML in Healthcare

AI is a comprehensive term that encompasses the emulation of human intelligence in computer systems programmed to imitate human actions [10]. ML, which falls under the umbrella of AI, can be further classified into supervised and unsupervised learning and can be applied to clinical datasets for creating robust risk models and redefining patients’ classifications [11].

Nowadays, algorithms have already demonstrated superior abilities to detect malignant tumors compared to radiologists and have provided valuable guidance to researchers in constructing cohorts for expensive clinical trials [12]. The most intricate form of ML is deep learning, which encompasses neural network models with multiple levels of features to predict outcomes. These models can unveil thousands of hidden features, due to the accelerated processing capabilities of modern graphics processing units and cloud architectures [13].

**Fig. 1:**
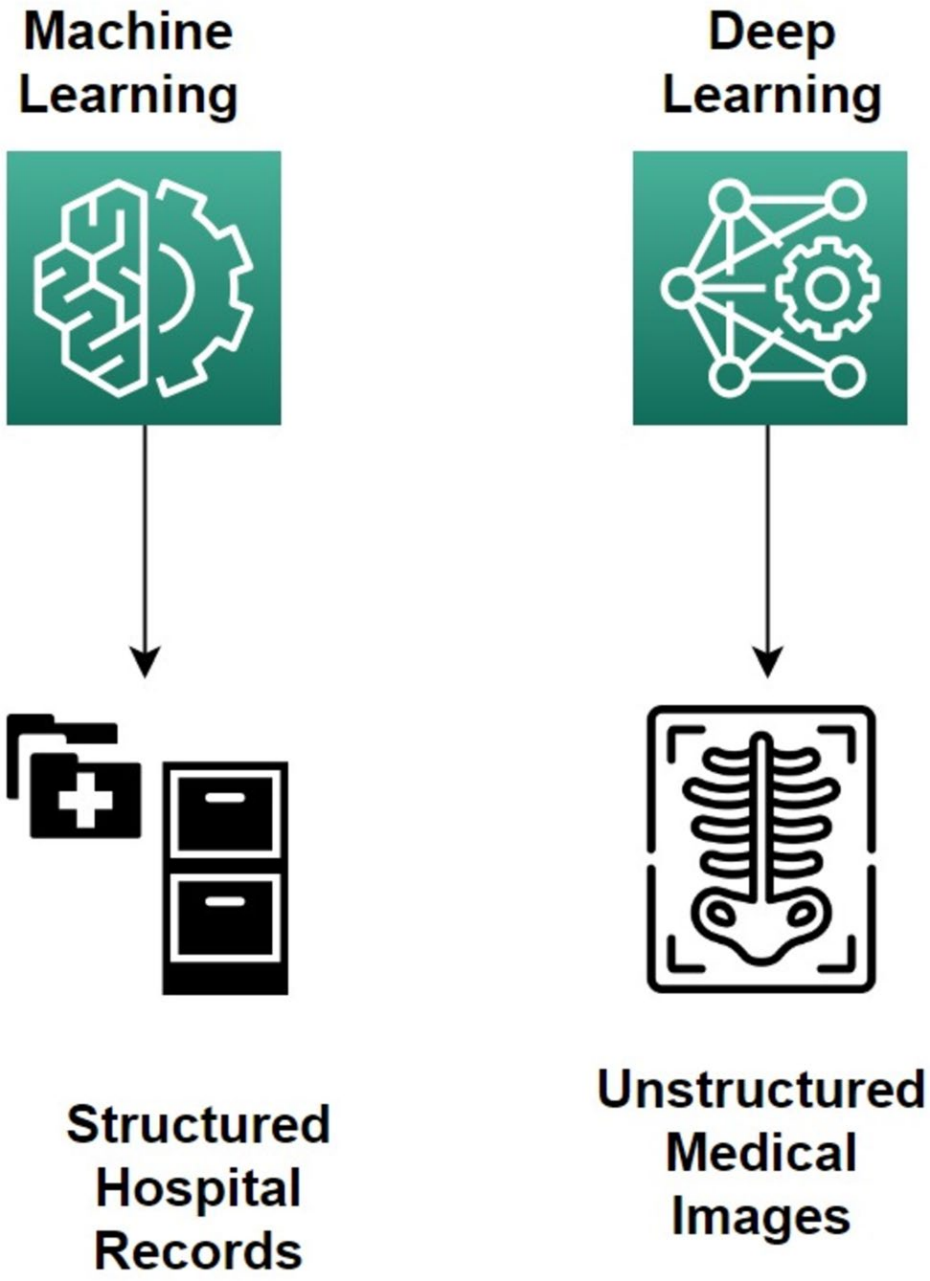
Machine learning vs. deep learning applications in healthcare

The application of deep learning in radiomics, which involves detecting clinically relevant features in imaging data beyond the capabilities of human visual perception, is becoming increasingly prevalent [14]. Figure 1 illustrates the types of application and medical inputs used in deep learing vs. machine learning. Despite accurate predictions, integrating AI-based diagnosis and treatment recommendations into clinical workflows and electronic health record (EHR) systems can present challenges at times.

### AI and ML in Prehospital Emergency Care

ML and AI applications facilitated the accurate prediction of outcomes that may be challenging for other risk-predicting tools to comprehend. Artificial neural networks (ANNs) have been employed to stratify the risk of intricate conditions like syncope in Emergency Care Departments (ED). ANNs are advantageous in this context due to their capability to evaluate complex and non-linear relationships between feature predictors and clinical outcomes [15, 16].

To date, only a limited number of studies have investigated the use of specific ML algorithms for predicting outcomes such as ICU admission or mortality in COVID-19 patients. Considering the potential value of ML-based decision rules and the critical nature of the pandemic, a collaborative endeavor is underway to identify the most effective ML applications for different datasets and diseases [17, 18].

Following the implementation of initiatives aimed at enhancing early recognition, dispatch protocols, bystander action, activation, and post-resuscitation care, several countries have witnessed an increase in survival rates after Out-of-Hospital Cardiac Arrest (OHCA) [19, 20]. In August 2018, a machine learning model was integrated into clinical practice at Copenhagen emergency medical services to enhance the recognition of OHCA. This model analyzed the conversation between the dispatcher and the caller, assisting the dispatcher in real-time to identify OHCA during the conversation. From September 2018 to December 2019, the machine learning model alerted dispatchers when an emergency call indicated a high probability of ongoing OHCA [21, 22].

### AI Applications in Prehospital Emergency Care

An automated external defibrillator (AED) is a portable device that delivers the heart with electric shocks in cases of SCA to regain normal rhythm. The European Society for Cardiology (ESC) and European Resuscitation Council (ERC) advocate for the widespread adoption of AEDs by both emergency services and non-medical staff to minimize the time taken for defibrillation [23]. The combination of AED and AI has the potential to further enhance the effectiveness of AEDs and improve outcomes for people experiencing sudden cardiac arrest. One of the applications is improved detection of shockable rhythms that can cause patients’ death if an electrical shock is not immediately delivered [24]. Figuera et al. developed an ML model for the detection of shockable rhythms in AEDs based on surface ECGs and OHCA data which mimics the real-life scenario of the use AEDs [25•].

Previous literature supports the advantages of employing ML models in resuscitation. Specifically, a deep learning model has been developed to achieve more accurate predictions of cardiac arrest and acute respiratory failure in intensive care units, outperforming the National Early Warning Score (NEWS) and the Modified Early Warning Score (MEWS) [26, 27]. The superiority of this deep learning model was attributed to its ability to detect relationships between vital signs and the ability to identify features with high importance and contribution to predicting risk. Despite ML demonstrating superior performance compared to the existing track‐and‐trigger systems, it required the utilization of a greater number of variables for feature learning [28].

### The Current State of the Field and Knowledge Gaps

AI has been explored for assessing the quality of CPR during resuscitation efforts. Computer vision algorithms can analyze video data from CPR training sessions or real-life events to provide real-time feedback to rescuers, helping them maintain correct compression depth, rate, and recoil [29]. A pilot study compared augmented reality (AR) CPR training with a standard audio-visual (AV) feedback manikin for healthcare providers and showed similar overall post-simulation CPR quality compared to standard AV feedback CPR training in healthcare providers [30]. In addition, AI algorithms have shown potential in automatically detecting cardiac arrest events in audio and video recordings. This can aid in faster recognition of cardiac arrest incidents and prompt initiation of CPR and AED use [31]. Not to mention the ability of AI in identifying areas with a higher likelihood of cardiac arrest incidents. This information can be used to improve emergency response planning and allocate resources more effectively and optimize the placement and distribution of AEDs in public spaces by analyzing historical cardiac arrest data, population density, and other factors [32–34].

While AI has shown promise in various aspects of healthcare, including medical imaging and diagnosis, its application in CPR and cardiac arrest management had some gaps and challenges. One of these challenges is the inability to adapt quickly to changes in patients’ conditions and provide real-time feedback to medical staff to adjust and enhance CPR [35, 36]. Also, integrating AI systems with existing AEDs and emergency response infrastructure requires careful consideration of data formats, communication protocols, and regulatory compliance [37].

### AI-based Prognostic Models in Post-resuscitation Phase

**Fig. 2:**
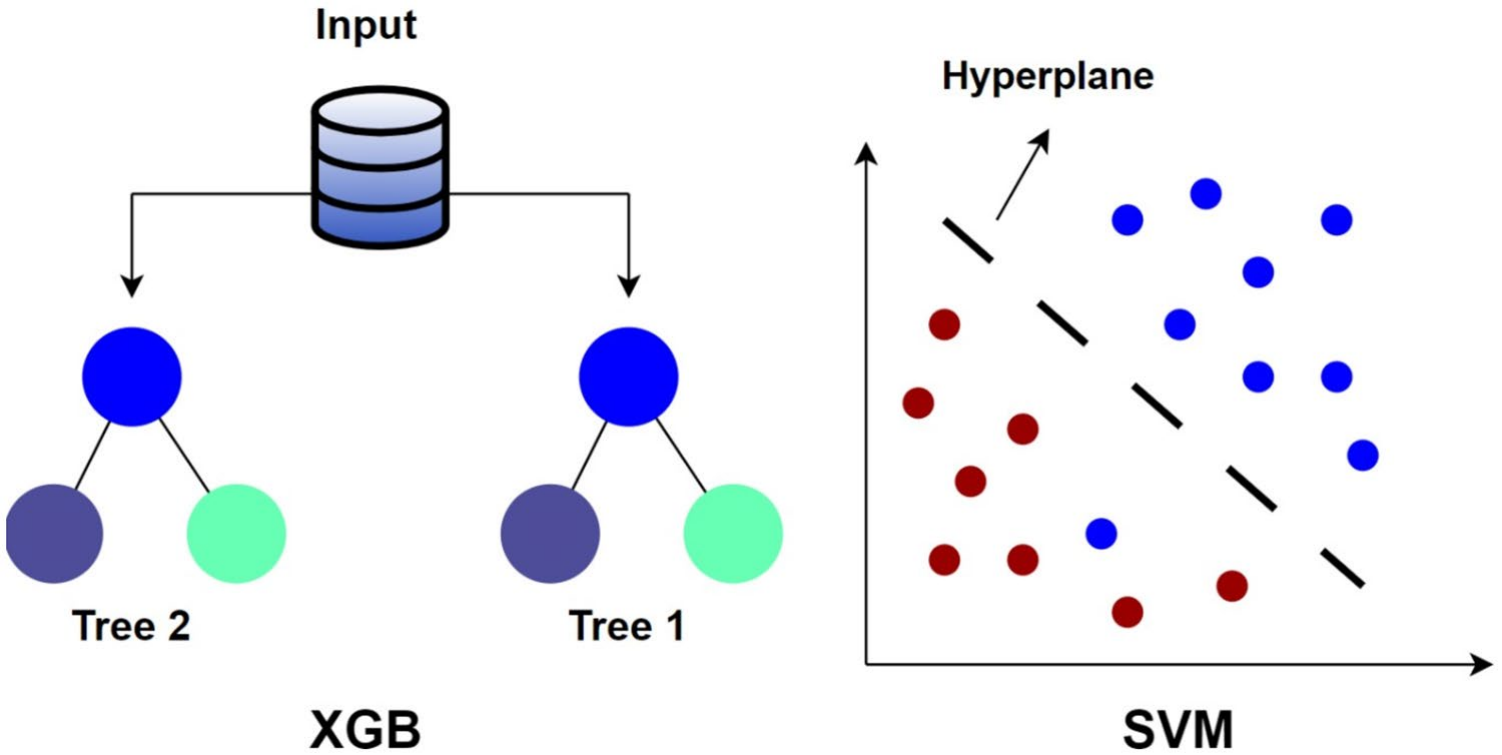
XGB vs. SVM model architecture

Cheng et al. proposed an ML-based model to predict the 30-day survival rate and survival-to-discharge rate after cardiac arrest of 1071 patients, showing the highest accuracy of 0.87 and 0.83 using the extreme gradient boosting (XGB) model compared to support vector machine (SVM) and logistic regression (LR) [38]. XGB models are a form of decision trees–based models, by combining decision tree models, where trees are incrementally included in the ensemble and trained to rectify the prediction errors of the preceding models. While SVM models implement a hyperplane that can distinctly group and classify samples [39]. Figure 2 shows the differences between XGB and SVM models. Harford et al. used embedded fully convolutional neural networks (EFCN) model to predict outcomes of survival for 2639 out-of-hospital cardiac arrests with 0.83 sensitivity using 27 features [40].

### Use of AI in Predicting Neurological Outcomes After Resuscitation

Kawai et al. implemented an AI-based prognostic model for the prediction of neurological outcomes after 3 h of resuscitation from 321 cardiac arrest patients using CT images, showing better performance than the previous gray-to-white matter ratio (GWR) in terms of precision-recall which accounts for the false-positive predictions, but comparable in terms of area under receiver operating characteristics curve (AUROC) [41]. Mansour et al. established a transfer learning approach to detect hypoxic–ischemic brain injury (HIBI) following cardiac arrests. They used CT scans of normal findings, to detect the outcome on follow-up scans, suggesting that the progression of HIBI can be accurately identified by AI in the early initial scan [42]

### AI in Cardio-Oncology Cardiac Arrests

With the co-prevalence of cardiovascular disease and cancer, cardio-oncology is expected to increase due to a globally aging population, and cardiac arrest, the second leading cause of medical death, is likely to be affected [43]. It is expected that the disparities in cardiac arrest observed in the general population would likely persist in the subgroup of patients with active cancer. However, the scarcity of research in this area has hindered effective efforts to address these ongoing disparities. A study by Monlezun et al. conducted a comprehensive analysis of cardio-oncology cardiac arrests, combining clinical, cost, and ethical aspects to improve effectiveness and cost-efficacy in healthcare systems. They developed a cardiac arrest risk prediction score for patients with cancer and introduced a novel clinical predictive model called The Cardiac Arrest Cardio-Oncology Score (CACOS), which can aid in the early prediction and improve resource allocation and health outcomes [44].

Utilizing AI to analyze cardiovascular data obtained from various diagnostic tests has shown promising results in accurately and inexpensively identifying cardiovascular risk, enabling early detection and intervention in cancer patients at risk for cardiovascular complications [45]. This approach has the potential to provide preventive and therapeutic opportunities in cardio-oncology, leading to better patient outcomes [46].

### The Use of AI in Defibrillators

In clinical practice, both external defibrillators (EDs) and implantable cardioverter defibrillators (ICDs) employ shock advisory algorithms to determine whether an electrocardiogram (ECG) tracing represents a shockable or non-shockable rhythm. Recently, the use of ML algorithms has been evaluated for shock decision classification, demonstrating a growing level of accuracy in this important task [47]. ICDs are used in individuals at high risk of sudden cardiac death. The crucial factor in delivering an appropriate and potentially life-saving shock from the ICD or AED lies in identifying a shockable rhythm, such as ventricular fibrillation (VF) and ventricular tachycardia (VT) [48]. AI has the potential to make a significant impact by decreasing the time it takes to deliver a shock and enhancing the efficiency of identifying shockable rhythms. This could lead to saving more lives by ensuring timely and appropriate interventions in cases of ventricular fibrillation and ventricular tachycardia.

Research on shock decision algorithms has been framed traditionally as a VF detection problem. Subsequently, ML algorithms such as support vector machines or ensemble methods effectively merged systematic and comprehensive extraction of EKG features with the selection of the most suitable feature subsets for ventricular fibrillation (VF) detection [49].

### Limitations of AI in Defibrillators

One of the limitations of AI solutions is that they necessitate large, annotated datasets to fine-tune the numerous trainable network parameters, often numbering in the thousands or even millions. Unfortunately, there is a scarcity of quality-controlled rhythm annotations in out-of-hospital cardiac arrest (OHCA) data. In addition, the use of AI in medical devices, including defibrillators, is subject to rigorous regulatory oversight to ensure safety and efficacy. Complying with these regulations can be time-consuming and resource-intensive [50]. Also, AI models can be vulnerable to adversarial attacks, where maliciously crafted inputs can lead to incorrect or harmful decisions. Securing AI algorithms in defibrillators from potential attacks is essential to maintain patient safety.

## Conclusion

In conclusion, SCAs remain a significant global cause of mortality, highlighting the critical need for effective interventions such as cardiopulmonary resuscitation (CPR) and electrical defibrillation. Despite advances in first responder systems, survival rates after out-of-hospital cardiac arrest (OHCA) remain low, and a substantial number of administered shocks do not achieve the desired outcomes. This calls for innovative approaches to improve resuscitation and defibrillation success rates. The application of AI in prehospital emergency care has shown promise in detecting shockable rhythms, predicting resuscitation success, and enhancing CPR quality through real-time feedback. AI’s potential extends to predicting neurological outcomes after resuscitation and even addressing cardio-oncology cardiac arrests, improving risk prediction and resource allocation.

However, AI in defibrillators also faces limitations, including the need for large, annotated datasets, scarce quality-controlled rhythm annotations, regulatory challenges, and vulnerability to adversarial attacks. Future studies are needed to address data quality and biases, advance the interpretability of AI models, and ensure robust security measures. Moreover, further research is needed to validate and integrate AI-based models into clinical workflows and medical devices effectively. Collaboration between researchers, healthcare providers, and regulatory bodies is essential to harness AI's potential fully and drive continuous improvements in cardiac arrest management, ultimately leading to better patient outcomes and increased survival rates.

## Data Availability

Data is derived from published studies.
